# Arabic translation and psychometric validation of the Problem-Solving Decision-Making scale: A cross-sectional study in Saudi Arabia

**DOI:** 10.1371/journal.pone.0349678

**Published:** 2026-05-22

**Authors:** Nouf Sahal Alharbi, Nouf Nasser Alsaheil, Jwaher Haji Alhaji, Sara Mohammed Karsha

**Affiliations:** 1 Department of Health Administration, College of Business Administration, King Saud University, Riyadh, Kingdom of Saudi Arabia; 2 Department of Health Sciences, College of Applied Studies and Community Service, King Saud University, Riyadh, Kingdom of Saudi Arabia; 3 Depatment of Public Relations, College of Applied Medical Sciences, King Saud University, Riyadh, Kingdom of Saudi Arabia; University of Hafr Al-Batin, SAUDI ARABIA

## Abstract

The Problem-Solving Decision-Making scale was originally developed in English to assess patient preferences across three domains: mortality, morbidity, and quality of life. This study aimed to translate, culturally adapt, and psychrometrically validate an Arabic version of the Problem-Solving Decision-Making scale using a sample of 505 adults from the general Saudi population. The translation followed World Health Organization guidelines for instrument translation and validation. Exploratory and confirmatory factor analyses were conducted to examine the underlying factor structure. Internal consistency was assessed using Cronbach’s alpha, while construct validity was evaluated through average variance extracted and the Fornell–Larcker criterion. Measurement invariance across sex was examined using multigroup confirmatory factor analysis. The Arabic version demonstrated strong psychometric properties. Exploratory factor analysis identified a four-factor structure explaining 56% of the variance, which showed good model fit (χ²/df = 3.22, CFI = 0.991, TLI = 0.990, SRMR = 0.070, RMSEA = 0.081). All factors exhibited satisfactory internal consistency. Convergent and discriminant validity were supported. Measurement invariance was established across sex, and latent mean comparisons indicated that females scored significantly higher than males across all four factors. These findings support the reliability and validity of the Arabic Problem-Solving Decision-Making scale and highlight its suitability for assessing patient involvement preferences in Arab population.

## Introduction

In recent decades, the relationship between healthcare providers and patients has undergone a fundamental transformation in its nature and the roles of its parties [1]. Historically, health decision-making has gradually shifted from the paternalistic model, in which physicians have a high level of responsibility to make decisions on behalf of their patients’ health, to more collaborative models that emphasis patient autonomy and the right to actively participate in decisions related to their health care [2–4]. This shift has been driven by several interrelated factors, including information revolution, ethical developments in modern medicine, an increasing focus on patient rights, and social and cultural changes that have reinforced the concept of individual autonomy in making health-related decisions [5–6]. In this context, patient involvement in medical decision-making has become one of the key components of patient-centred healthcare models, in which patients are viewed as active partners in the decision-making process rather than passive recipients of health care [7,8].

This shift towards greater patient participation has been associated with in health outcomes including healthcare quality, patient satisfaction and treatment adherence [9,10]. Adopting a shared decision-making approach between physicians and patients contributes to improving patient satisfaction with the health care they receive, enhancing their adherence to the treatment plan, and increasing their sense of empowerment in managing their health condition [8,11,12]. In addition, several researchers have indicated that patient involvement in decision-making may help improve communication between healthcare providers and patients and assist in aligning treatment decisions with patients’ values and personal preferences, which is a key element in providing high-quality health care centred on the needs and expectations of the patients [13,14].

As patient involvement becomes more central to healthcare decision-making, understanding how patients engage with health information and treatment options, as well as the factors influencing their level of participation, is essential [15]. With the rapid advances in modern medical fields, patients face increasing amounts of complex health information that may include sensitive or unexpected results, sometimes exceeding the scope of the primary research question. This may raise a number of ethical and social issues related to how these results are shared with patients or research participants and the possibility of misinterpreting this information or its effects on social and personal lives [16]. Accordingly, patients are no longer viewed as passive recipients of medical decisions; rather, they are viewed as essential partners in the decision-making process, contributing to the discussion of available treatment options and assessing their benefits and risks in light of their personal preferences, health, and social conditions [3,4]. This shift has led to increased research interest in studying patients’ preferences for their role in medical decision-making and understanding the factors that may influence these preferences, which is considered a fundamental step towards enhancing shared decision-making models and improving the quality of health care provided to patients [17–19].

Although the role of the patient in decision-making has been increasingly emphasised, the literature indicates that medical participation does not follow a single fixed pattern, but is characterised by a high degree of variation according to patients’ preferences and their expected roles in the treatment process [20,21]. Although modern health care encourages shared decision-making, patients differ in how they prefer to engage. Some patients prefer to actively participate in discussing treatment options and choosing the final decision. Others tend to delegate the decision to healthcare providers, whereas others prefer a middle model represented by shared decision-making between doctors and patients [22,23].

In this context, patient participation is conceptualised as a spectrum ranging from a passive role, in which the patient delegates the decision entirely to the doctor, to an active role, in which the patient participates in evaluating treatment options and making the final decision, whereas shared decision-making represents a balance between these two patterns [24,25]. Emanuel and Emanuel described this variation through different models of the doctor–patient relationship, including the paternalistic model, where the doctor retains authority, and the informative patient model, where the patient takes a larger role, in addition to interpretive and deliberative models that emphasise dialogue and alignment with patients’ values and preferences [26]. Within this framework, shared decision-making has become one of the most accepted approaches in modern health care

However, patients’ preferences for participation are not fixed across all clinical situations, but may vary depending on the nature of the health decision. Some decisions are preference-sensitive, where multiple treatment options exist without a clearly superior choice, making patient values central to determining the most appropriate option [25]. In contrast, in urgent or life-threatening situations that require rapid intervention, patient involvement may be more limited, relying more on the clinician’s expertise [27]. A systematic review has indicated that characteristics, such as clinical urgency, available alternatives, and associated risks, influence how decision-making is shared [25].

In addition, patients’ preferences are not shaped only by medical information but are also influenced by individual and contextual factors. Demographic characteristics, such as age, sex, education, and socioeconomic status, may affect patients’ willingness to participate and their level of health knowledge [28,29]. Cultural and religious backgrounds also play an important role, particularly in societies where social and religious values influence health behaviours and perceptions of decision-making. System-level factors, including the nature of the healthcare system and the patient–provider relationship, further contribute to this variability [16,27,30,31]. In light of this, models of patient participation in decision-making cannot be viewed as a unified model applicable across all contexts, but should instead be understood within a range of influencing factors. Therefore, understanding the variation in patient preferences for participation is essential for improving healthcare systems and delivering more patient-centred care. Accordingly, identifying and accurately measuring patient preferences for participation is a critical step in understanding decision-making processes and guiding the selection of appropriate assessment tools [22,28,29].

In response to this complexity, increasing attention has been directed towards the developing tools that measure patients’ preferences for medical decision-making [32,33]. A wide range of these instruments has been developed internationally, including the Health Opinion Survey, Clinical Decision-Making Scale, Autonomy Preference Index, and Control Preference Scale [34–37]. These instruments have been widely used to assess patients’ roles in clinical decision-making; however, many of them extend beyond decision-making preferences alone and simultaneously capture patients’ preferences for receiving health-related information. As highlighted in prior systematic reviews, this dual focus reflects two overlapping but conceptually distinct domains, information-seeking and decision-making, which are often measured concurrently within the same instrument [33]. However, most of these tools have been developed within Anglo-American cultural contexts, reflecting implicit assumptions about the nature of the relationship between doctors and patients. This raises fundamental questions regarding the suitability of these tools for application in different cultural contexts, especially given the limited number of cross-cultural validation tests that assess their stability and validity outside their original settings. Moreover, many of these measures have not undergone a comprehensive psychometric evaluation when used in diverse communities, which may affect the accuracy of the measurement and interpretation of the results [32,33].

This gap becomes even more evident in the Arab context, where the use of tools to assess decision-making preferences remains limited, both in terms of the number of available instruments and the level of validation. Although efforts have been made to translate existing tools, they remain scarce and are often confined to linguistic adaptation or preliminary validation, without comprehensive psychometric evaluation that adequately accounts for cultural and contextual factors [38,39]. Considering this limitation, some studies in the region have developed their own instruments rather than adopting existing standardised measures. For example, a study conducted in the United Arab Emirates utilised a questionnaire developed based on prior theoretical concepts without using a standardised measurement tool [40]. The validation of this instrument was limited to face and content validity, without performing a comprehensive psychometric analysis. Importantly, the findings of this study highlight the need to distinguish between patients’ preferences related to problem-solving and those related to decision-making, two conceptually distinct dimensions that are often combined in traditional instruments. Accordingly, the authors recommended the use of instruments capable of capturing this distinction and ensuring their applicability across different cultural contexts [40], specifically recommending the use of Problem-Solving Decision-Making (PSDM) scale [41].

In this context, the PSDM scale is considered a prominent conceptual tool for assessing patients’ preferences regarding their roles in health-related decision-making. It is distinguished by its ability to differentiate between two fundamental dimensions that are often conflated in traditional instruments: the problem-solving dimension, which involves identifying health problems and exploring treatment alternatives, and the decision-making dimension, which pertains to selecting the final treatment option [15,41]. The scale is grounded in a theoretical framework that conceptualises health decision-making as a multistage process rather than a single event, wherein roles are distributed between physicians and patients to varying degrees [42]. This PSDM scale has been widely applied across a range of healthcare contexts and populations. Its use has been well documented in chronic conditions, including cardiovascular and hospitalised patient populations [15,43,44], and in mental health settings, such as anxiety, depression, and perinatal mental health [45,46]. In addition, this scale has been used in reproductive health contexts, including fertility treatment and contraceptive decision-making [47,48]. Furthermore, the scale has been adapted and used in general population samples [49], supporting its applicability to diverse populations. Despite its widespread use, evidence regarding the psychometric properties of the PSDM scale remains limited, particularly in terms of its systematic and cross-cultural validation [32]. In many instances, the scale has been applied in its original form, without a comprehensive evaluation of its validity and reliability across diverse linguistic and cultural settings. Furthermore, translated versions remain scarce and are often supported by limited psychometric assessment [49]. However, the evidence regarding the structural validity of the PSDM scale remains inconsistent, with variations reported across studies and limited confirmatory analyses [32,33,49].

This gap clearly underscores the need for rigorous studies aimed at culturally adapting and systematically validating the scale to ensure its suitability in non-Western contexts. In Saudi Arabia, this need is particularly important given the ongoing transformation of the healthcare system under Vision 2030 and the introduction of a Model of Care that emphasises patient-centred care and shared decision-making [50]. Understanding patient preferences and roles in decision-making is essential to support this transformation and ensure alignment with the evolving healthcare delivery model. Accordingly, this study aimed to translate, culturally adapt, and psychometrically validate the PSDM scale into an Arabic context. This study further examined convergent and discriminant validity and the underlying factor structure of the scale and assessed measurement invariance across sexes to ensure reliability and applicability across different population groups. This study aimed to provide a robust and contextually appropriate tool for assessing patient preferences in decision-making within the healthcare systems in the region.

## Materials and methods

### Instrument

The PSDM scale was developed by Kraetschmer in 1994 in Canada and originally administrated in English to assess patient preferences regarding their involvement in health treatment decision-making [41]. The tool derived from Debr and Baumann’s model of patient participation distinguishes the choice behaviour (clinical reasoning) into two aspects: problem-solving, which refers to ‘identifying the most accurate answer to a problem’, and decision-making, which refers to ‘selecting from multiple possible options, often involving compromises or trade-offs’ [42]. This scale is a validated instrument that contained 18 items comprising three vignettes: the morbidity scenario (‘Suppose you often experience a burning sensation when you go to the bathroom. You usually have to push to begin to urinate, and sometimes, dribbling occurs after urination’); the mortality scenario (‘Suppose you had mild chest pains for 3 days and decided that you should visit your doctor about this’); and the quality-of-life scenario (‘Suppose you and your partner have been trying for pregnancy but have been unsuccessful for more than a year’).

Each scenario presents two tasks: problem-solving and decision-making. The problem-solving scale includes the following questions: ‘Who should determine (diagnose) the likely cause of your symptoms?’ (Diagnosis); ‘Who should determine the treatment options?’ (Options); ‘Who should determine the risks and benefits of each treatment option?’ (Risks and benefits); and ‘Who should determine the likelihood that each of these risks and benefits will occur?’ (Probability). The decision-making scale includes the following questions: ‘Given the risks and benefits of the possible treatments, who should decide how acceptable these risks and benefits are for you?’ (Utility); ‘Given all the information about the risks and benefits of the possible treatments, who should decide which treatment option should be selected?’ (What is done?). The participants were instructed to provide their answers on a 5-point Likert scale: ‘1 (doctor alone), 2 (mostly the doctor), 3 (doctor and you equally), 4 (mostly you), and 5 (you alone)’ [41].

The PSDM scale was conceptually grounded in Deber and Baumann’s theory of patient participation, which distinguishes between two elements of choice behaviour: problem-solving (identifying the most accurate or correct answer) and decision-making (selecting the most desired outcome based on patient preferences) [21]. The original version demonstrated acceptable internal consistency, with Cronbach’s alpha values ranging from 0.71 to 0.90 across the three vignettes. The scale was subsequently used in later studies, where the items were operationally grouped into two subscales representing problem-solving and decision-making. In 2010, Dehlendorf et al. adapted the instrument by structuring the problem-solving and decision-making components into two separate sets of questions, treating them as distinct operational groupings consistent with the original conceptual framework, but without conducting a comprehensive psychometric validation of the underlying factor structure [48]. In the translated Portuguese version, exploratory analysis using principal component analysis (PCA) initially identified three underlying components. However, to maintain conceptual consistency with the original theoretical model, the authors subsequently imposed a two-factor solution in line with prior literature [49], while reporting higher internal consistency, with Cronbach’s alpha values ranging from 0.82 to 0.95. Recent systematic reviews evaluating instruments designed to assess patient preferences in decision-making have further highlighted that the PSDM scale has largely relied on conceptual assumptions regarding its factor structure, with limited evidence of rigorous structural validation, including confirmatory factor analysis and measurement invariance testing across populations. These findings underscore the need for further psychometric investigation to examine the underlying factor structure of the scale across diverse cultural contexts [32,33].

### Translation of the Problem-Solving Decision-Making scale

We followed the World Health Organization’s guidelines for translating instruments from English into other languages [51]. Initially, the scale developer was contacted to obtain permission to translate the scale into Arabic and validate it with the Saudi population. The forward translation of the PSDM scale was independently performed by three native Arabic speakers fluent in English. This step was important to ensure that the translation considered the local understanding of medical terms. After completing the translation, the Arabic and original versions were compared by an expert committee comprising professionals from diverse fields, including clinical practice, health policy, and linguistics. The committee reviewed the forward-translated version to ensure conceptual and semantic equivalence with the original scale. The development of the first Arabic version of the PSDM scale took over 2 weeks.

Next, the Arabic version was back-translated by a professional translator who is a native Arabic speaker and proficient in English. The translator was blinded to the original PSDM. The original, translated, and back-translated versions were reviewed by an expert committee to identify and resolve any discrepancies in meaning. Finally, the translated instrument was used to conduct a pilot study with a group of 18 individuals from the general Saudi population to verify the accuracy of the translation and ensure that it was understandable. The final Arabic version of the PSDM is presented in S1 File.

### Validation in the general Saudi population

This cross-sectional study was conducted with a convenience sample of 505 Saudi Arabians from Riyadh City, with participants at least 19 years of age. Prior to data collection, ten data collectors were trained in the study protocol, including the use of the instrument, standardised interviewing techniques, and informed consent documentation procedures, according to scientific and ethical standards. Data were collected between 9 February 2024 and 18 April 2024. Ten data collectors were assigned to different areas of Riyadh, the capital city of Saudi Arabia, because of its social and economic diversity. According to city main areas, two data collectors were assigned to each area, and each data collector was required to collect data from approximately 50 participants who were recruited from public places, such as malls, parks, and offices in Riyadh. Before presenting the survey to each participant, the data collectors read a standardised consent script explaining the purpose of the study, voluntary nature of their participation, and confidentiality of the data provided. The participants were then shown the same text on the tablet used for the survey and indicated their agreement by selecting ‘Yes, I agree to participate’. Data collectors were supervised throughout the data collection period to ensure that the data collection process was executed correctly.

In terms of sample size, Bartlett indicated that to perform PCA, the minimum size required is five times the number of items; accordingly, a sample size of 90 was required for the current study [52]. Nunnally suggested that the sample size should not be < 300 [53]. However, our convenience sampling method resulted in 505 participants responding, and verbal informed consent was obtained from all participants.

Regarding ethical concerns, no personal information that could identify the participants (such as names, national identification numbers, or other identifiers) was collected during this study. To ensure the full protection of data privacy, data were recorded anonymously. In addition, the data were secured, with access provided only to the principal investigator, and the overall approach to data processing and analysis was in keeping with recent recommendations for privacy protection in behavioural and psychological research [54]. The King Saud University Scientific Research Ethics Committee (KSU-HE-23–878) reviewed and approved the study protocol and informed consent procedures.

### Statistical analyses

Data were analysed in RStudio, using the ‘lavaan’, and ‘SemTools’ packages. Frequencies and percentages were calculated to explore participants’ demographic characteristics. Prior to performing statistical analyses, the dataset was cleaned to ensure quality and completeness. Missing responses were excluded from the final analyses. Exploratory factor analysis (EFA) and confirmatory factor analysis (CFA) were performed to explore the latent factor structure of the Arabic PSDM scale. The total number of participants (505) was randomly divided into two independent groups: one-third for EFA (170) and the remaining two-thirds (337) for CFA. Sampling adequacy and sphericity were assessed using the Kaiser–Meyer–Olkin (KMO) measure and Bartlett’s test of sphericity to ensure data applicability for EFA. A KMO value > 0.60 indicates an adequate sampling adequacy for factor analysis. The internal structural validity of the EFA was then examined using the principal component extraction method with varimax rotation, and only factor loadings > 0.4 were retained for interpretation. For validation purposes, an additional oblique (Promax) rotation was performed to examine the inter-factor correlations and confirm the suitability of the orthogonal rotation method. Therefore, the Varimax rotation was retained for interpretation. In addition, a parallel analysis was performed to determine the optimal number of factors to be retained by comparing the observed eigenvalues with those from the simulated datasets. Factors were retained when the observed eigenvalues exceeded the simulated ones [55].

CFA was performed on the second subsample to evaluate the fit of both the original hypothesised factor structure and the factor structure derived from the EFA. Given the ordinal nature of the item responses, model estimation was performed using the weighted least squares mean and variance-adjusted (WLSMV) estimator [56]. Model fit was assessed using multiple fit indices, including the chi-squared statistic relative to degrees of freedom (χ²/df), comparative fit index (CFI), Tucker–Lewis index (TLI), standardised root mean square residual (SRMR), and root mean square error of approximation (RMSEA). Acceptable model fit was indicated by χ²/df values < 5, CFI and TLI values of ≥ 0.90, SRMR values < 0.08, and RMSEA values < 0.08 [57]. The composite reliability (CR) and average variance extracted (AVE) were calculated to assess convergent validity. CR values ≥ 0.70 and AVE values ≥ 0.50 were considered indicative of adequate convergent validity. Discriminant validity was evaluated using the Fornell–Larcker criterion, whereby the square root of the AVE for each construct was compared with inter-factor correlations. Discriminant validity was supported when the square root of the AVE exceeded the corresponding inter-factor correlations [58]. The internal consistency reliability of the scale was examined using a full sample (n = 505) by assessing Cronbach’s alpha and item correlations. Cronbach’s alpha values of ≥ 0.70 were considered acceptable [59]. Corrected item–total correlations of ≥ 0.30 and factor loadings of ≥ 0.40 were deemed adequate for item retention [59,60].

A multigroup CFA was performed to assess measurement invariance across sexes using the WLSMV estimator. Configural, metric, and scalar invariance models were sequentially tested to examine the equivalence of factor structures across males and females. Configural invariance was first assessed by allowing all parameters to be freely estimated across groups. Metric invariance was then evaluated by constraining factor loadings to equality, followed by scalar invariance in which item intercepts were constrained across sex groups. Measurement invariance was evaluated based on changes in the model fit indices, with ΔCFI values < 0.01 indicating invariance [61]. After establishing scalar invariance, the latent mean differences between males and females were examined, with males specified as the reference group [62]. Standardised factor loadings were also estimated separately for each sex to evaluate the stability of item–factor relationships across groups. All invariance analyses were performed using the CFA subsamples.

## Results

### Sociodemographic characteristics

As shown in Table 1, almost half of the participants were female (53.9%), and 42.4% were aged between 30 and 39 years. Most participants were married (57.4%) and had a bachelor’s degree (58.6%). Regarding occupational status, 70.7% of the participants were either employed or retired.

**Table 1:** Characteristics of the participants (no. [%], n = 505).

Sex	
Male	233 (46.1%)
Female	272 (53.9%)
**Age (years)**	
20–29	155 (30.7%)
30–39	214 (42.4%)
40–49	95 (18.8%)
50–59	26 (5.1%)
≥ 60	15 (3%)
**Marital status**	
Married	290 (57.4%)
Single	183 (36.2%)
Divorced	24 (4.8%)
Widowed	8 (1.6%)
**Educational level**	
No formal education	1 (0.2%)
High school or less	68 (13.5%)
Diploma	54 (10.7%)
Bachelor’s	296 (58.6%)
Postgraduate	86 (17%)
**Occupation**	
Student	54 (10.7%)
Unemployed	94 (18.6%)
Employed/retired	357 (70.7%)

### Factor analysis

**Fig 1:**
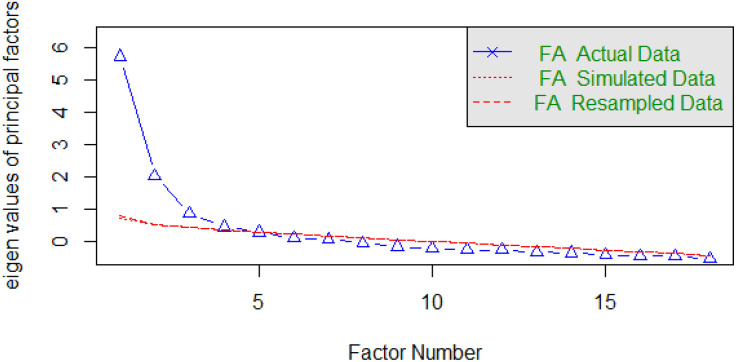
Parallel Analysis Scree Plot for the Arabic PSDM Scale.

**Fig 2:**
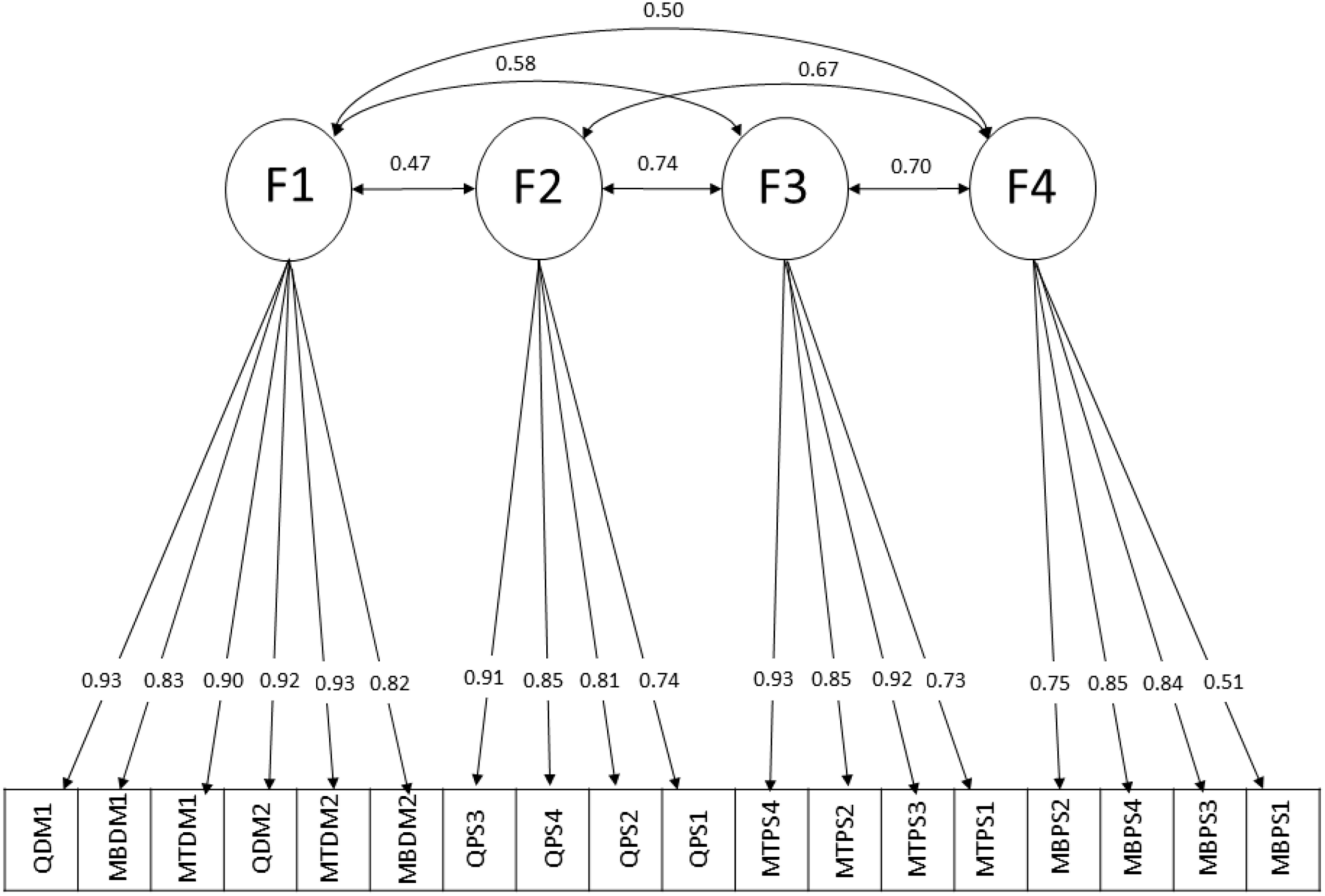
Standardized factor loadings for the four-factor PSDM model obtained from confirmatory factor analysis. Note. Q = Quality of Life; MB = Morbidity; MT = Mortality. DM1 = Utility; DM2 = What is done. PS1 = Diagnosis; PS2 = Options; PS3 = Risk and benefits; PS4 = Probability.

Prior to factorial analysis, the feasibility of the scale was evaluated using the KMO and Bartlett’s tests, which showed good values (KMO = 0.80, p < 0.001). Using the method of eigenvalues > 1, PCA with the varimax rotation method identified four factors, with a total explained variance of 56.1%. The 18 items were divided into the four factors as follows: factor 1 (Utility and ‘What is done’ questions of mortality, morbidity, and quality-of-life scenarios); factor 2 (Diagnoses, Options, Risks and benefits, and Probability questions of quality-of-life scenario); factor 3 (Diagnoses, Options, Risks and benefits, and Probability questions of mortality scenario); and factor 4 (Diagnoses, Options, Risks and benefits, and Probability questions of morbidity scenario) (Table 2). This factor structure is illustrated in a scree plot (Fig 1) that shows a clear inflection point after the fourth factor. This indicates that the four factors explain most of the total variance in the data. Consistently, the first four factors had eigenvalues > 1, supporting the four-factor solution. Furthermore, the eigenvalues of the parallel analysis exceeded the raw simulated data set in the first, second, third, and fourth factors (6.28 vs. 0.51, 2.07 vs. 0.35, 0.82 vs. 0.30, and 0.45 51 vs. 0.25, respectively), indicating a four-factor structure of the Arabic PSDM scale (Table 3). Furthermore, the results of the CFA indicated that the original two-factor model demonstrated poor fit to the data (CFI = 0.80, TLI = 0.77, RMSEA = 0.102, 90% confidence interval [CI] = 0.095–0.109, χ²/df = 6.25), suggesting that the hypothesised structure did not adequately represent the underlying constructs in this sample. In contrast, the four-factor model showed substantially improved fit across all indices (CFI = 0.991, TLI = 0.990, RMSEA = 0.081, 90% CI = 0.073–0.090, χ²/df = 3.22), with acceptable residual fit (SRMR = 0.070 for both models) (Table 4). The four-factor structure is illustrated in the CFA path diagram (Fig 2). To further evaluate the dimensional structure of the scale, a second-order confirmatory factor analysis was conducted to examine the presence of a higher-order construct. The model demonstrated acceptable fit (CFI = 0.99, TLI = 0.99, RMSEA = 0.074). However, the four-factor model remained the preferred representation of the data.

**Table 2:** Rotated factor matrix for the Arabic version of the PSDM scale using Varimax rotation (n = 170).

Item	Factor			
	1	2	3	4
Utility (quality of life)	.831			
Utility (morbidity)	.800			
Utility (mortality)	.788			
What is done (quality of life)	.722			
What is done (mortality)	.718			
What is done (morbidity)	.657			
Risk and benefits (quality of life)		.813		
Probability (quality of life)		.742		
Options (quality of life)		.589		
Diagnoses (quality of life)		.440		
Probability (mortality)			.747	
Options (mortality)			.732	
Risk and benefits (mortality)			.584	
Diagnoses (mortality)			.442	
Options (morbidity)				.608
Probability (morbidity)				.558
Risks and benefits (morbidity)				.482
Diagnoses (morbidity)				.409

**Table 3:** Parallel analysis of raw data eigenvalues and simulative data eigenvalues (n = 170).

Factor	Raw data eigenvalues	Simulative data eigenvalues
**1**	6.28	0.51
**2**	2.07	0.35
**3**	0.82	0.30
**4**	0.51	0.25
**5**	0.20	0.19
**6**	0.05	0.14

**Table 4:** Comparison of model fit indices between the original two-factor and four-factor CFA models (n = 335).

	Four-factor model	Two-factor model
χ²/df	3.22	6.25
CFI	0.991	0.80
TLI	0.990	0.772
SRMR	0.070	0.070
RMSEA [90% CI]	0.081 [0.073–0.090]	0.102 [0.095–0.109]

### Construct validity

The construct validity of the four-factor model was examined using CR, AVE, and the Fornell–Larcker criterion. All constructs demonstrated CR values > 0.70 and AVE values > 0.50, indicating adequate convergent validity. Discriminant validity was established across all constructs, as the square root of the AVE for each construct exceeded the corresponding inter-factor correlations. These findings further support the adequacy of the four-factor model (Table 5).

**Table 5:** Construct validity of the four-factor model.

Construct	CR	AVE	√AVE	F1	F2	F3	F4
**F1**	0.92	0.80	0.89	**0.89**			
**F2**	0.84	0.70	0.84	0.47	**0.84**		
**F3**	0.86	0.75	0.86	0.58	0.74	**0.86**	
**F4**	0.74	0.57	0.75	0.50	0.67	0.70	**0.75**

### Internal consistency

The internal consistency coefficient, calculated using Cronbach’s alpha coefficients, ranged from 0.87 to 0.90 across factors. The mean inter-item correlation ranged from 0.38 to 0.80, with only one item having a value < 0.4 (Diagnosis question for morbidity vignette) which remained close to the acceptable threshold (Table 6). In addition, Cronbach’s alpha was calculated for the problem-solving and decision-making scales of the instrument and for each component identified by the PCA. The problem-solving and decision-making scales, along with all four components, showed good internal consistency (Cronbach’s alpha = 0.90, 0.91, 0.86, 0.83, and 0.73, respectively) (Table 7).

**Table 6:** Items and scale correlations (n = 505).

	Mean inter-item correlation	Corrected item-total correlation	Cronbach’s alpha if item is deleted
**Problem-solving scale**			
**Morbidity scenario**	0.430		
Diagnosis		0.388	0.898
Options		0.519	0.889
Risks and benefits		0.645	0.881
Probability		0.643	0.881
**Mortality scenario**	0.608		
Diagnosis		0.578	0.885
Options		0.694	0.880
Risks and benefits		0.706	0.879
Probability		0.712	0.879
**Quality-of-life scenario**	0.562		
Diagnosis		0.543	0.887
Options		0.588	0.885
Risks and benefits		0.697	0.879
Probability		0.643	0.882
**Decision-making scale**			
**Morbidity scenario**	0.669		
Utility		0.734	0.902
What is done		0.726	0.903
**Mortality scenario**	0.753		
Utility		0.729	0.902
What is done		0.766	0.897
**Quality-of-life scenario**	0.795		
Utility		0.807	0.891
What is done		0.788	0.894

**Table 7:** Cronbach’s alphas for the scale and its components (n = 505).

Variables	Items (n)	Cronbach’s alpha
Problem-solving (all scenarios)	12	0.892
Problem-solving (morbidity scenario)	4	0.739
Problem-solving (mortality scenario)	4	0.860
Problem-solving (quality-of-life scenario)	4	0.836
Decision-making (all scenarios)	6	0.914
Decision-making (morbidity scenario)	2	0.800
Decision-making (mortality scenario)	2	0.858
Decision-making (quality-of-life scenario)	2	0.886
Problem-solving and decision-making (morbidity scenario)	6	0.761
Problem-solving and decision-making (mortality scenario)	6	0.834
Problem-solving and decision-making (quality-of-life scenario)	6	0.827
Problem-solving and decision-making (all scenarios)	18	0.907

### Measurement invariance across sex

To examine measurement invariance across sexes, a series of increasingly restrictive models were tested, including configural, metric, and scalar invariance. The model fit indices for each step are listed in Table 8. Configural invariance was first assessed by allowing all parameters to be freely estimated across males and females while maintaining the same factor structure. The model demonstrated good fit (CFI = 0.993, TLI = 0.991, RMSEA = 0.064), indicating that the overall factor structure was equivalent across sex groups. Metric invariance was tested by constraining the factor loadings to be equal across the groups. The change in model fit indices was negligible (ΔCFI = 0.001), supporting metric invariance across sex groups and indicating that factor loadings were comparable between males and females. Scalar invariance was subsequently evaluated by constraining the item intercepts across groups. The model showed minimal changes in fit indices (ΔCFI = 0.001, RMSEA = 0.061), supporting scalar invariance and suggesting that the scale operated equivalently across sex groups.

**Table 8:** Measurement invariance across sex (WLSMV) estimator.

Model	CFI	TLI	RMSEA	ΔCFI
Configural	.993	.991	.064	–
Metric	.992	.990	.065	.001
Scalar	.993	.992	.061	.001

Standardised factor loadings according to sex ranged from 0.44 to 0.94 for females and from 0.60 to 0.95 for males, indicating generally high and comparable loadings across groups (Table 9). Given that scalar invariance was established, the latent mean differences between males and females were examined. Male patients were used as the reference group. As shown in Table 10, females demonstrated significantly higher latent means across all four factors than males: factor 1 (estimate = 0.759, SE = 0.274, p = 0.005), factor 2 (estimate = 1.004, SE = 0.338, p = 0.003), factor 3 (estimate = 0.840, SE = 0.287, p = 0.003), and factor 4 (estimate = 0.285, SE = 0.123, p = 0.021).

**Table 9:** Standardised factor loading according to sex.

Factor	Item	Female	Male
Factor 1	Utility (quality of life)	0.94	0.92
Factor 1	Utility (morbidity)	0.79	0.88
Factor 1	Utility (mortality)	0.91	0.89
Factor 1	What is done (quality of life)	0.94	0.90
Factor 1	What is done (mortality)	0.92	0.95
Factor 1	What is done (morbidity)	0.81	0.84
Factor 2	Risk and benefits (quality of life)	0.90	0.94
Factor 2	Probability (quality of life)	0.85	0.86
Factor 2	Options (quality of life)	0.90	0.94
Factor 2	Diagnoses (quality of life)	0.72	0.76
Factor 3	Probability (mortality)	0.93	0.93
Factor 3	Options (mortality)	0.84	0.87
Factor 3	Risk and benefits (mortality)	0.91	0.94
Factor 3	Diagnoses (mortality)	0.75	0.74
Factor 4	Options (morbidity)	0.68	0.83
Factor 4	Probability (morbidity)	0.82	0.88
Factor 4	Risks and benefits (morbidity)	0.84	0.86
Factor 4	Diagnoses (morbidity)	0.44	0.60

**Table 10:** Latent mean differences across sexes.

Factor	Estimate (reference male)	SE	p-value
Factor 1	0.759	0.274	0.005
Factor 2	1.004	0.338	0.003
Factor 3	0.840	0.287	0.003
Factor 4	0.285	0.123	0.021

## Discussion

We translated the PSDM scale from English to Arabic using a validated translation process, considering the linguistic and cultural differences between the two languages, because English is an Indo-European language and Arabic is a Semitic language [63]. We adopted a rigorous methodology to ensure the accuracy and cultural relevance of our translation. The psychometric properties of the Arabic version of the scale were tested by administering it to 505 Saudi individuals. Upon comparing the distribution of demographic characteristics with the latest national population statistics [64,65], the sample appeared to be generally representative of the population. Therefore, the results provide strong evidence of the scale’s quality and reliability, reflecting the rigour and accuracy of our translation and adaptation methodology.

The reliability of the Arabic version of the PSDM scale was assessed by interpreting the Cronbach alpha values, which ranged between 0.73 and 0.91, indicating good internal consistency across the scale components. This not only underscores the tool’s consistency in capturing individuals’ unique ways of problem-solving and decision-making but also strengthens the reliability of the Arabic PSDM scale. These findings are consistent with the original English and Portuguese versions of the PSDM scale, both of which reported high internal consistency across scale components [41,49]. This robust internal consistency enhances the effectiveness of the tool in evaluating decision-making preferences and problem-solving approaches among patients and the general population. Understanding these personal strategies is essential for designing tailored interventions.

Regarding the instrument’s structure, unlike the original English and translated Portuguese versions, which supported a two-component conceptualisation of problem-solving and decision-making [41,49], the Arabic version retained decision-making as a single factor and further divided problem-solving into three factors: mortality problem-solving, morbidity problem-solving, and quality-of-life problem-solving, resulting in a four-factor structure. Although the RMSEA value for the four-factor model was at the upper bound of acceptable fit, RMSEA is sensitive to model complexity and large sample sizes. When considered alongside the excellent CFI, TLI, and SRMR values, the overall pattern of fit indices supports the adequacy and superiority of the four-factor model over the original two-factor structure. these findings should also be interpreted in light of the prior literature which suggests that the factor structure of the PSDM scale has largely been conceptually assumed rather than empirically established through rigorous confirmatory factor analysis [32,33]

Construct validity analysis further supported the four-factor structure of the Arabic PSDM scale. All constructs demonstrated adequate convergent validity, with the CR and AVE values exceeding the recommended thresholds. Discriminant validity was confirmed using the Fornell–Larcker criterion, which indicates that the four factors represent distinct but related constructs. These findings provide additional evidence for the structural validity of the adapted model. Convergent validity was further examined to evaluate the coherence of items within each factor. The results indicate adequate convergence across the four factors, suggesting that the items within each domain are strongly related and reflect the same underlying constructs. This pattern supports the conceptual distinction between the problem-solving and decision-making components while maintaining internal consistency within each scenario. Taken together, these findings provide additional evidence for the structural validity of the Arabic PSDM scale and are consistent with the adequacy of the four-factor model.

Measurement invariance across sexes was established, supporting the equivalence of the factor structure between males and females and allowing a meaningful comparison of latent means. Following this, females demonstrated higher scores than males across all PSDM factors, suggesting potential differences in preferences for participation in decision-making rather than measurement bias. Among the studies using the PSDM scale, one reported higher participation preferences among females [43], whereas other applications of the instrument did not explicitly examine sex differences [15,44–46]. Beyond studies using the PSDM scale, systematic reviews investigating decision-making preferences have reported inconsistent findings, with no clear or consistent sex-related patterns across populations and instruments. These reviews suggest that preferences for participation in decision-making may depend on contextual factors, including the healthcare setting and decision characteristics [25,30,32]. Furthermore, cultural background is an important influence on patients’ participation preferences, indicating that the variability in sex-related findings may be shaped by sociocultural factors [33]. Evidence from Arab populations using different decision-making instruments supports this interpretation. Studies conducted in Arabic-speaking settings have reported higher participation preferences among females compared with males [40,66]. Taken together, these findings suggest that sex differences in decision-making preferences reflect cultural and contextual influences rather than universal patterns.

This interpretation can be better understood within the specific cultural context of the study population. The Kingdom of Saudi Arabia is an Islamic country and is home to Islam`s holiest sites. Therefore, religion is likely to influence individuals’ interpretations of their health experiences and engagement in problem-solving practices [67,68]. From a religious perspective, Pargament et al. [69] ranged problem-solving practices from completely relying on God to self-reliance or cooperative practices. This framework suggests that religious beliefs shape how individuals approach health-related decisions, including diagnostic and treatment choices. Supporting this interpretation, religious and spiritual beliefs can influence participation in healthcare decision-making and shape individuals’ preferences for involvement in treatment decisions [16,69]. Moreover, a recent systematic review examining patient autonomy in non-Western contexts identified cultural, social, and religious factors as key influences shaping autonomous decision-making processes in healthcare settings [70]. Another review focusing on Muslim patients highlighted that religious and cultural values were central to the healthcare experience and should be considered when supporting patient-centred and shared decision-making [68]. In our study, the emergence of four factors may reflect the influence of religious and cultural factors on how individuals approach their diagnostic and treatment decisions. Nonetheless, the decision-making domain remained unified across scenarios, which is consistent with the theoretical framework of shared decision-making. This variation does not undermine the applicability of the scale; rather, it highlights the importance of contextual adaptation in psychometric evaluations. Therefore, cultural differences, including potential religious influences, might have contributed to the modified structure of the Arabic version of the PSDM scale.

In contrast, the separation observed in the problem-solving domain may be explained by differences in the nature and complexity of clinical scenarios. A systematic review identified that decision characteristics, such as uncertainty, multiple options, trade-offs, and the impact of the decision, varied across clinical contexts and levels of severity. These characteristics correspond to the core elements of problem-solving, including evaluating options, weighing risks and benefits, and considering potential outcomes. Therefore, differences among mortality, morbidity, and quality-of-life scenarios may lead individuals to engage in problem-solving processes differently, resulting in distinct factor structures across scenarios [25]. Furthermore, a previous study using the PSDM instrument across diverse patient populations has shown that preferences for involvement in healthcare decisions vary depending on the clinical context, suggesting that the perceived seriousness and consequences of a condition can influence how individuals approach decision-related tasks [15]. These findings provide a possible explanation of the scenario-specific structures observed in this study. However, these factors are hypothetical and require further investigation.

This study provides healthcare organisations with a reliable instrument for assessing patients’ decision-making and problem-solving practices in Arabic-speaking countries. This represents an important development, especially under the current National Health Transformation Program and the enhancement of value-based healthcare, as the framework promotes patient-centred care and the alignment of decisions with patient preferences and values. Understanding patients’ problem-solving and decision-making processes will also help organisations develop training programs and interventions to enhance decision-making processes in alignment with the needs of the population. The knowledge generated from the Arabic PSDM scale can be integrated into clinician training programs to enhance shared decision-making competencies, inform organisational policies that emphasise patient engagement, and guide the development of tailored interventions that integrate care and patient values. These strategies can support the 2030 objectives of the Saudi Health Transformation Program and broaden the shift towards value-based health care.

Despite the valuable insights this study provides regarding the translated scale, acknowledging its limitations when interpreting the results is important. Although this study included a considerable sample of 505 participants with varied demographic characteristics, the findings may be less applicable to populations with lower educational backgrounds. The sample consisted mostly of highly educated individuals, with 58.6% holding a bachelor’s degree and 17% holding a postgraduate degree. This educational profile may have influenced their familiarity with health decision-making and problem-solving concepts. Therefore, future studies should include participants from a wider range of educational levels to enhance the generalisability of the findings. In addition, criterion validity was not assessed in this study, as no external validated instruments measuring the same construct were included in the questionnaire. Future research is recommended to examine criterion validity by comparing the Arabic PSDM scale with other established measures of patient decision-making preferences. Finally, although CFA demonstrated an acceptable overall model fit, some fit indices were marginal. Given the sensitivity of the model fit indices, particularly RMSEA, to sample size and model complexity, future studies using larger and more diverse samples are recommended to further confirm the factorial structure and enhance the stability of the measurement model.

## Conclusions

This study translated and psychometrically validated the PSDM scale into Arabic for use in the general Saudi population. The findings support a four-factor structure and demonstrate satisfactory reliability and structural validity of the Arabic version. To the best of our knowledge, this is the first study to provide a comprehensive psychometric validation of the Arabic PSDM scale, including confirmatory factor analysis, convergent validity, and measurement invariance across sex, supporting comparability between males and females.

The emergence of a four-factor structure, rather than the original two-factor model, suggests that the conceptualization of problem-solving and decision-making may vary across cultural contexts. This modified structure does not contradict the theoretical foundations of the original scale but may reflect contextual differences in how individuals approach health-related decisions. The separation of problem-solving factors across scenarios may indicate variations in perceived severity and decision urgency among mortality, morbidity, and quality-of-life contexts.

Although cultural and religious influences may contribute to these differences, religiosity was not directly measured in the present study. Future research should incorporate measures of religious and spiritual practices, such as Salat al-Istikharah, to examine their potential influence on individuals’ approaches to problem-solving and health-related decision-making. Additionally, further studies using larger and more diverse samples, including participants from other Arab countries, are recommended to enhance generalizability and further validate the factor structure across different population

## Supporting information

S1 File Arabic version of the PSDM scale. (PDF)
