# Supplementary material for: Arabic translation and psychometric validation of the Problem-Solving Decision-Making scale: A cross-sectional study in Saudi Arabia
Source: PLoS One. 2026 May 22;21(5):e0349678. doi: 10.1371/journal.pone.0349678 (PMC13196962; doi:10.1371/journal.pone.0349678)
Supplement: S1 File — (PDF) [file pone.0349678.s001.pdf]

Problem-Solving and Decision-Making Scale
Forward Translation

**We will present you with three cases. Please answer each case by selecting the appropriate choice.**
**Case One (morbidity Case): Suppose you often feel a burning sensation when you go to the bathroom. Usually, you need to push to start urinating, and sometimes dribbling occurs after urination.**
1- Who should determine (diagnose) the possible cause of your symptoms?
- The doctor alone
- Mostly the doctor
- The doctor and I together
- Mostly me
- Myself alone
2- Who should determine what the treatment options are?
- The doctor alone
- Mostly the doctor
- The doctor and I together
- Mostly me
- Myself alone
3- Who should determine what the risks and benefits are for each treatment option?
- The doctor alone
- Mostly the doctor
- The doctor and I together
- Mostly me
- Myself alone
4- Who should determine how likely each of these risks and benefits are to occur?
- The doctor alone
- Mostly the doctor
- The doctor and I together
- Mostly me
- Myself alone
5- Considering the risks and benefits of these proposed treatments, who should decide how acceptable these risks and benefits are for you?
- The doctor alone
- Mostly the doctor
- The doctor and I together
- Mostly me
- Myself alone
6- Considering all the information about the risks and benefits of the proposed treatments, who should decide which treatment option should be chosen?
- The doctor alone
- Mostly the doctor
- The doctor and I together
- Mostly me
- Myself alone
**Case Two (mortality Case): Suppose you have had mild chest pain for 3 days and decided you should visit your doctor about this pain.**
1- Who should determine (diagnose) the possible cause of your symptoms?
- The doctor alone
- Mostly the doctor
- The doctor and I together

- Mostly me
- Myself alone
2- Who should determine what the treatment options are?
- The doctor alone
- Mostly the doctor
- The doctor and I together
- Mostly me
- Myself alone
3- Who should determine what the risks and benefits are for each treatment option?
- The doctor alone
- Mostly the doctor
- The doctor and I together
- Mostly me
- Myself alone
4- Who should determine how likely each of these risks and benefits are to occur?
- The doctor alone
- Mostly the doctor
- The doctor and I together
- Mostly me
- Myself alone
5- Considering the risks and benefits of these proposed treatments, who should decide how acceptable these risks and benefits are for you?
- The doctor alone
- Mostly the doctor
- The doctor and I together
- Mostly me
- Myself alone
6- Considering all the information about the risks and benefits of the proposed treatments, who should decide which treatment option should be chosen?
- The doctor alone
- Mostly the doctor
- The doctor and I together
- Mostly me
- Myself alone
**Case Three (Quality of Life case): Suppose you and your spouse have been trying to conceive for more than a year but have not succeeded.**
1- Who should determine (diagnose) the possible cause of your symptoms?
- The doctor alone
- Mostly the doctor
- The doctor and I together
- Mostly me
- Myself alone
2- Who should determine what the treatment options are?
- The doctor alone
- Mostly the doctor
- The doctor and I together
- Mostly me
- Myself alone
3- Who should determine what the risks and benefits are for each treatment option?
- The doctor alone
- Mostly the doctor
- The doctor and I together
- Mostly me

- Myself alone
4- Who should determine how likely each of these risks and benefits are to occur?
- The doctor alone
- Mostly the doctor
- The doctor and I together
- Mostly me
- Myself alone
5- Considering the risks and benefits of these proposed treatments, who should decide how acceptable these risks and benefits are for you?
- The doctor alone
- Mostly the doctor
- The doctor and I together
- Mostly me
- Myself alone
6- Considering all the information about the risks and benefits of the proposed treatments, who should decide which treatment option should be chosen?
- The doctor alone
- Mostly the doctor
- The doctor and I together
- Mostly me
- Myself alone
